# Time over minimum inhibitory concentration and variations in beta-lactam concentrations during prolonged and intermittent infusions in patients with septic shock: A post hoc observational study

**DOI:** 10.1177/03000605261457800

**Published:** 2026-07-14

**Authors:** Ylva Mattisson, Jonas Tverring

**Affiliations:** 1 Division of Infection Medicine, Department of Clinical Sciences, 5193 Lund University, Sweden; 2 Department of Infectious Diseases, Helsingborg Hospital, Sweden

**Keywords:** Septic shock, therapeutic drug monitoring, beta-lactam antibiotics, continuous renal replacement therapy, target attainment

## Abstract

**Objective:**

Critically ill patients with septic shock frequently exhibit variability in antibiotic pharmacokinetics. We aimed to investigate the antibiotic concentration and time over minimum inhibitory concentration in patients with septic shock treated with prolonged and intermittent infusions.

**Methods:**

We performed a post hoc analysis of a prospective study of patients with septic shock admitted to the intensive care unit at Helsingborg hospital from 2016 to 2018. Plasma samples were collected every 4 h for the initial 72 h following intensive care unit admission. We analyzed samples for cefotaxime (intermittent dosing), piperacillin–tazobactam (prolonged infusion), and meropenem (prolonged infusion) concentrations. Minimum inhibitory concentration values determined using epsilometer tests of bloodstream pathogens were collected, and corresponding epidemiological cut-off values were used for target attainment calculations. The primary outcome was percentage time over minimum inhibitory concentration or four times the minimum inhibitory concentration. We used linear mixed effects models for analyzing repeated measures of continuous variables.

**Results:**

In total, 21 patients met the criteria for septic shock, and a total of 341 samples were analyzed (average 16 per patient). All patients achieved concentrations above the minimum inhibitory concentration for 100% of the time, and 83% of the patients achieved concentrations above 4 times the minimum inhibitory concentration for 100% of the time. We found an association between continuous renal replacement therapy and levels of antibiotics exceeding the upper therapeutic target (*p* = 0.021, odds ratio: 15, 95% confidence interval: 1 to 974).

**Conclusion:**

Target attainment was high in this cohort study of critically ill patients with septic shock, with all patients achieving beta-lactam concentrations over the minimum inhibitory concentration 100% of the time.

## Introduction

Septic shock remains a significant global health concern and a leading cause of morbidity and mortality worldwide.^1,2^ Treatment of septic shock relies on prompt antimicrobial therapy.^3^ Beta (ß)-lactams are the most used class of antibiotics in critically ill patients. They exhibit a time-dependent killing pattern, where the duration of exposure to antibiotics is important for their effectiveness.^4,5^ ß-lactams are generally known for their wide therapeutic index and few adverse effects; however, the increased use of therapeutic drug monitoring (TDM) has revealed abnormally high or low concentrations in several studies.^6,7^ This entails a risk of suboptimal therapy,^6–9^ potential toxicity,^10^ and an increase in antibiotic resistance.^4,11^ Critically ill patients with septic shock are heterogeneous in age and comorbidities and often experience cardiovascular, liver, and renal organ dysfunction,^12^ which contribute to pharmacokinetic (PK) variability.^13,14^ Dosing guidelines for adults are often extrapolated from studies on middle-aged healthy volunteers.^4^ Several intensive care units (ICUs) have adopted continuous or extended infusion regimens instead of traditional intermittent dosing to optimize treatment.^3^ Although extended or continuous infusions are theoretically appealing compared with intermittent dosing, their superiority seems difficult to prove with certainty or the benefit is small.^15^ There may be added complicating factors associated with the use of continuous infusions such as intra-individual PK variability over time and durability or decomposition in vials.^16^ We hypothesized that the unbound concentrations of ß-lactams in the blood of patients in the ICU with septic shock display important intra- and interindividual differences despite the use of prolonged infusions. We aimed to determine what proportion of patients with septic shock in the ICU attain an unbound ß-lactam concentration above the minimum inhibitory concentration (MIC) (free drug time over MIC (fT) > MIC) or four times the MIC (fT > 4x MIC) for 100% of the study period.

## Methods

### Study design and population

This post hoc*,* retrospective, cohort study was conducted at the ICU of Helsingborg Hospital. Helsingborg emergency hospital is in southern Sweden and has an ICU with seven beds. The hospital serves an estimated population of 250,000. Study participants were recruited from September 2016 to February 2018 for an original study that aimed to investigate the prognostic performance of repeated measurements of heparin-binding protein (HBP) in the plasma, the results of which were published in 2020.^17^ The Swedish Ethical Review Authority approved the study design of the original study in 2016 (Dnr 2016/271) and this post hoc analysis in 2020 (Dnr 2021–02692). Patients were evaluated for eligibility on arrival to the ICU by the attending intensive care physician. The inclusion criteria were as follows: (a) age ≥18 years and (b) suspected septic shock. Septic shock was suspected in patients with an acute increase of ≥2 points in the total sequential organ failure assessment (SOFA) score due to a suspected infection in addition to a need for vasopressors to maintain a mean arterial pressure (MAP) ≥65 mmHg despite adequate fluid resuscitation and a serum lactate level of ≥2 mmol/L.^18^ No exclusion criteria were applied. Patients meeting the inclusion criteria were invited to participate in the study and asked to provide written informed consent. When patients were unable to provide consent, their next-of-kin was approached for permission. For the post hoc antibiotic concentration analysis, patients were excluded if insufficient samples were available or if MIC or epidemiological cut-off values (ECOFFs) data necessary for target attainment calculations could not be obtained. Patients included in the study received treatment with either cefotaxime given as intermittent doses (20 min infusions), piperacillin–tazobactam as a prolonged infusion (3-h infusions) or meropenem as a prolonged infusion, depending on clinical presentation. The continuous renal replacement therapy (CRRT) modality was continuous venovenous hemodialysis (CVVHD) with Prismaflex using regional citrate or heparin anticoagulation with Phoxilium or Hemosol solutions. The reporting of this study conforms to the Strengthening the Reporting of Observational Studies in Epidemiology (STROBE) guidelines.^19^

### Data and sample collection

A trained ICU nurse prospectively collected data at the time of plasma sampling using a study-specific case report form. Demographic characteristics, medical history, laboratory and microbiology results, monitoring parameters, physiological variables, and survival data were retrospectively extracted from electronic health records. Comorbidities were classified using the Charlson Comorbidity Index (CCI).^20^ Renal function was assessed at the time of inclusion by determining the estimated glomerular filtration rate (eGFR) using serum creatinine values and the revised Lund–Malmö equation (LM-rev).^21^ Serial plasma samples were collected starting within 2 h of ICU admission and subsequently every 4 h for up to 72 h or until death or ICU discharge. Arterial blood was drawn into 2.7-mL sodium citrate tubes, centrifuged, and the plasma aliquoted and frozen at −80°C within 1 h.

### Analyses of ß-lactam concentrations

Samples from the HBP–Kinetics in Septic Shock (KISS) study were stored in a biobank at the Infection Clinic SUS Lund (BD-3) at −80°C for up to 5 years prior to analyses.^22^ Antibiotic concentration analyses were performed at the Clinical Chemistry Department in Lund using liquid chromatography–mass spectrometry (LC–MS) with a coefficient of variation <15%.^23^ An internal check was performed to compare analyses of sodium citrate and ethylenediaminetetraacetic acid (EDTA) plasma samples*.* Antibiotic concentrations were corrected for protein binding, and free concentrations were calculated. Concentrations of unbound piperacillin, cefotaxime, and meropenem were estimated to be 70%, 60%, and 98% of the total value, respectively.^24–26^ Tazobactam concentration was not analyzed. Epsilometer (E) tests were performed on saved samples from included patients unless an MIC value was already known and recorded in the database. The European Committee on Antimicrobial Susceptibility Testing (EUCAST) ECOFFs were used for all target attainment calculations, with individual MIC values presented alongside for reference. The toxicity analysis was restricted to piperacillin and meropenem, for which the upper therapeutic target thresholds were defined as 157 mg/L and 44 mg/L, respectively.^10,27–29^ However, there is no consensus, and some studies have suggested higher trough concentrations, especially for neurotoxic events.^30^ The threshold for determining toxic levels of cefotaxime is not defined, although the French Societies of Pharmacology and Therapeutics (SFPT) and Anesthesia and Intensive Care Medicine (SFAR) have suggested an upper threshold for optimal treatment of 60 mg/L for cefotaxime.^31,32^

### Outcome definition

*Primary outcome.* The primary outcome was the proportion of patients with unbound ß-lactam concentrations at or above MIC for 100% of the time (100% fT > MIC) or at or above 4 times the MIC (100% fT > 4x MIC) from the first to the last sampling time point (from ICU admission up to 72 h).

#### Secondary outcomes. *The following two secondary outcomes were recorded:*

- *Toxicity.* Occurrence of ß-lactam concentrations above the upper therapeutic target thresholds and possibly related adverse events;
- *Clearance*. Association between absolute unbound ß-lactam concentrations and individual eGFR or with use of CRRT.

### Statistical analyses

All patient data were deidentified prior to analysis. Time over MIC was calculated using linear interpolation between measured concentrations obtained at 4-h intervals over 72 h. The percentage of time above MIC (fT > MIC) was determined as the proportion of the total observation period, during which free antibiotic concentrations exceeded the MIC, calculated as the sum of time intervals with concentrations above MIC divided by the total observation time. In addition to analyzing time over MIC, we recorded whether concentrations reached the upper therapeutic target levels, as defined by specific thresholds. The association between antibiotic toxicity and individual patient factors was performed using univariate and multivariate logistic regression modeling. Fisher's exact test was used for the comparison of proportions with less than five observations, such as for assessing the correlation between upper therapeutic target levels and CRRT. The secondary analysis regarding clearance was performed using linear mixed effects regression modeling, adjusting for repeated measurements within individuals using an identity link. Missing data were handled using a complete case approach. Statistical analyses were conducted using Stata MP 18.0 and R (version 2023.12.1 + 402). No sample size calculation was performed for this post hoc study.

## Results

### Patient characteristics

**Figure 1.**
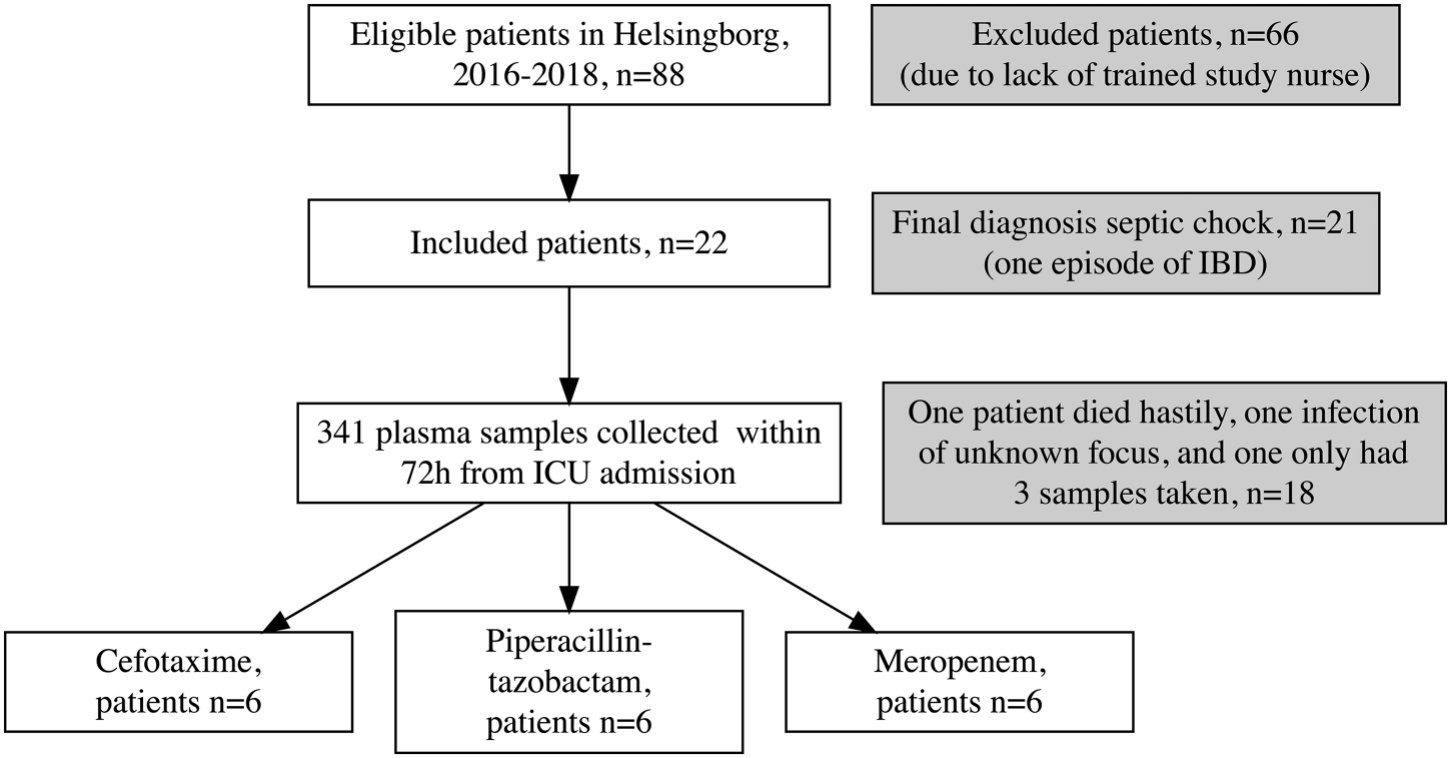
Flow chart depicting the selection process of eligible patients for the study conducted in Helsingborg from 2016 to 2018, along with subsequent exclusions and reasons.

During the original prospective study period, there were a total of 88 eligible patients, out of which 22 were included. The mean age of the 22 included patients was 66.5 (range: 34–84) years; 9 patients were female (41%), and 7 were surgical admissions (32%). The median Simplified Acute Physiology Score 3 (SAPS 3) score was 70 (range: 47–100) points, and the median SOFA score on the first day was 12.5 (range 6–18). Twenty-one out of the 22 patients received a final diagnosis of septic shock, whereas one patient (ID: 18) experienced circulatory failure due to a severe episode of inflammatory bowel disease. The 28-day mortality rate was 23% (n = 5), and the 90-day mortality rate was 27% (n = 6). Figure 1 illustrates the patient selection flowchart, and Table 1 includes information about patient characteristics.

**Table 1.** Patient characteristics.

	Age	Sex		Max SOFA		eGFR		Albumin level					
ID	*(years)*	*(M/F)*	CCI	*score*	CRRT	*mL/min/1.73 m^2^*	CKD stage	*g/L*	Type of infection	Microbiological findings	MIC/ECOFF	Antibiotic treatment	Dose
1	34	F	0	6	No	78	2		LRI	Negative	0.06^a^	CTX	1 g q8h^b^
2	64	M	9	13	Yes	17	5	27	IE	*S. aureus*	2 (4^a^)	CTX	1 g q8h
3	80	M	6	14	No	47	3		LRI	*Actinomyces, S. anginosus*	1 (1^a^)	PT	4 g q8h
4	73	M	4	16	Yes	24	4	30	Cholangitis	*E. coli, C. perfringens*	0.032 (0.06^a^)	MRN	1 g q8h
5	70	M	3	8	No	71	2		GI perf	Anaerobes	1^a^	PT	4 g q8h
6	83	M	6	15	No	53	3		LRI	Influenza A	2^a^	PT	4 g q8h
7	75	F	4	10	No	94	1	24	SSTI	*S. pyogenes*	0.032 (0.03^a^)	CTX	1 g q8h
8	52	M	1	18	Yes	22	4	31	Pharyngeal abscess	*S. pyogenes*	0.004 (0.008^a^)	MRN	1 g q8h
9	67	M	5	16	Yes	30	3	16	Cholangitis	*E. coli*	4 (8^a^)	PT	4 g q8h
10	81	M	5	11	No	51	3		Unknown	Negative	-	-	
11	57	F	2	13	No	63	2		UTI	*E. coli*	0.064 (0.25^a^)	CTX	1 g q8h
12	77	M	3	15	No	19	4		LRI	Negative	-	-	
13	65	M	5	13	Yes	32	3		LRI	*L. pneumophila*	-	CTX	2 g q8h^c^
14	63	M	5	8	No	93	1		LRI	*H. influenzae*	0.064 (0.06^a^)	CTX	1 g q8h
15	65	F	8	9	No	23	4		UTI	*E. coli*	8^a^	PT	4 g q8h
16	53	F	4	11	No	55	3		SSTI	Negative	0.125^a^	MRN	0.5 g q8h
17	84	F	7	12	No	66	2		SSTI	*S. dysgalactiae*	-	-	
18	66	F	3	9	No	15	1		No inf. (IBD)	Negative	-	-	
19	45	M	1	8	No	120	1	9.5	SSTI	*S. pyogenes*	0.004 (0.008^a^)	MRN	1 g q8h
20	73	F	6	15	No	69	2		LRI	Negative	2^a^	MRN	1 g q8h
21	67	M	4	11	Yes	19	5		GI perf	*K. pneumoniae, E. faecium*	2 (8^a^)	PT	4 g q8h
22	47	F	0	15	Yes	29	4		LRI	*S. pyogenes*	0.004 (0.008^a^)	MRN	1 g q8h
**Median (range)**	**67 (34–84)**		**4 (0–9)**	**12.5 (6–18)**		**52 (17–120)**		**24 (9.5–30)**					

a ECOFF; ^b^First dose: 2 g; ^c^After dose 8 lowered to 1 g q8h.

M: male; F: female; CCI: Charlson comorbidity index; SOFA: sequential organ failure assessment; eGFR: estimated glomerular filtration rate; CKD: chronic kidney disease; LRI: lower respiratory infection; IE: infectious endocarditis; GI perf: gastrointestinal perforation; SSTI: skin and soft tissue infection; UTI: urinary tract infection; ID: identification; No. inf: no infection; IBD: irritable bowel disease; MIC: minimum inhibitory concentration; ECOFF: epidemiological cut-off value; CTX: cefotaxime; PT: piperacillin–tazobactam; MRN: meropenem; CRRT: continuous renal replacement therapy; *E. coli: Escherichia coli; S. dysgalactiae: Streptococcus dysgalactiae; S. pyogenes: Streptococcus pyogenes; K. pneumoniae: Klebsiella pneumoniae; E. faecium: Enterococcus faecium; S. aureus: Staphylococcus aureus; S. anginosus: Streptococcus anginosus; C. perfringens: Clostridium perfringens; L. pneumophila: Legionella pneumophila; H. influenzae: Haemophilus influenza.*

### Sample, blood culture, and antimicrobial dosing characteristics

**Figure 2.**
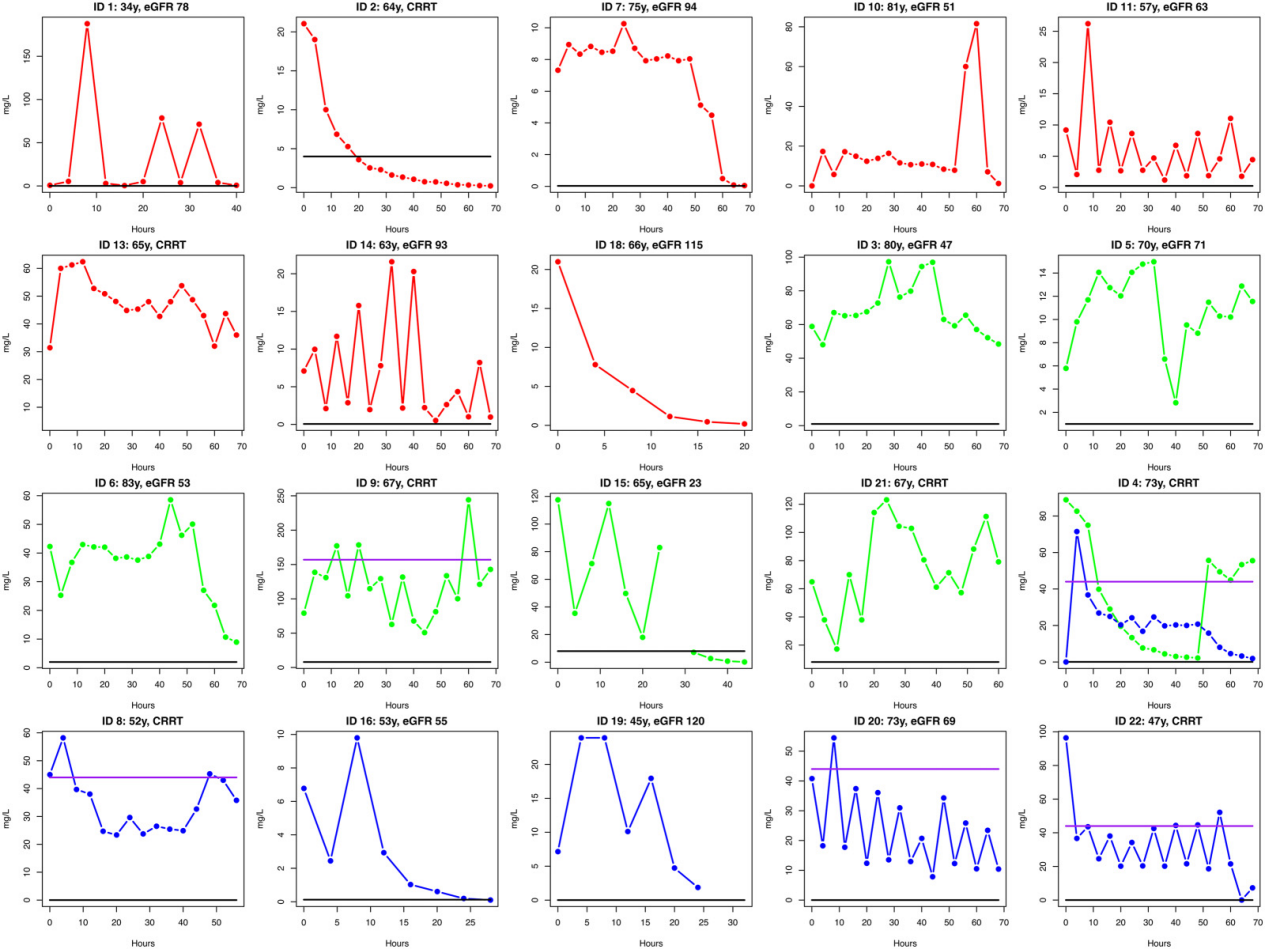
Graphs illustrating antibiotic concentrations over time for each patient. Cefotaxime was administered intermittently, whereas piperacillin–tazobactam and meropenem, administered as prolonged infusions. Line styles and point shapes for each antibiotic as well as for the epidemiological cut-off value (ECOFF) and the upper threshold for extended infusion are defined in the legend. Additional patient information can be found in Table 1.

In total, 341 plasma samples were collected. Thus, an average of 16 samples per patient were collected every 4 h during the first 3 days at the ICU. Four patients (IDs: 10, 12, 17, and 18) were excluded from the antibiotic concentration analysis. One patient (ID: 12) was excluded due to early death. Only three samples were collected from another patient (ID: 17) at the start of treatment. One patient (ID: 10) had an infection of unknown origin, whereas another (ID: 18) experienced acute severe exacerbation of irritable bowel disease, rendering analysis unfeasible due to the lack of MIC or ECOFF data. Patients undergoing cefotaxime treatment were administered intermittent doses, whereas those being administered piperacillin–tazobactam or meropenem received prolonged infusion. One patient (ID: 10) accidentally received four doses of cefotaxime at once. Five patients (IDs: 2, 15, 16, 18, and 19) received less than five doses of the ß-lactam antibiotics analyzed. Eleven patients had positive blood cultures (50%). The most common blood culture finding was *Escherichia coli* and *Streptococcus pyogenes,* accounting for 36% each out of positive blood cultures. In 16 patients (73%), cultures from other sites such as the respiratory tract, urinary tract, wound site, and abdominal abscess were positive. All identified pathogens were susceptible to the administered ß-lactam antibiotics. Figure 2 provides an overview of the antibiotic concentrations in individual patients over time compared with the MIC or ECOFF. Table 1 presents a list of estimated MICs.

### Primary outcome: Target attainment

All assessable patients (n = 18) achieved 100% fT >MIC. In total, 83% of patients achieved 100% fT >4x MIC (n = 15) (Table 2).

**Table 2.** Summary of results for primary and secondary outcomes.

Target attainment	Number of patients	% of total (n = 18)	Median MIC (range)
fT100% > MIC	18	100%	0.3125 (0.004–8)
fT100% > 4× MIC	15	83%	
fT50% > 4× MIC	17	94%	
Toxicity			*Correlation with toxicity*
Toxic levels	5	28%	
CRRT	4	22%	*p* = 0.021 (OR 15, 95% CI: 1 to 974)^a^
Adverse effects	2	11%	*p* = 0.043^b^
Clearance			*Concentration increase (mg/L)*
CRRT	7	39%	41.2 (*p* = 0.031, 95% CI: 7 to 76)
CKD stage			12.2 (*p* = 0.12, 95% CI: −3 to 28)^c^

Summary of results for primary and secondary outcomes. Toxicity defined as concentrations exceeding the upper therapeutic threshold (piperacillin >157 mg/L; meropenem >44 mg/L). ^a^Fisher's exact test; ^b^Fisher's exact test; ^c^CKD was analyzed as a continuous variable.

OR and CI not available due to infinite numbers.

MIC: minimum inhibitory concentration; CRRT: continuous renal replacement therapy; OR: odds ratio; CI: confidence interval; CKD: chronic kidney disease; fT: free drug time over MIC.

### Secondary outcomes

*Toxicity (piperacillin and meropenem).* In five patients (IDs: 4, 8, 9, 20, and 22), concentrations were intermittently above the upper therapeutic target levels for extended infusions. All these patients were on CRRT, and two eventually died. Both deceased patients (IDs: 9 and 20) had received CRRT due to renal failure. Two patients had documented adverse effects potentially attributable to ß-lactam antibiotics, including one with concentrations exceeding the upper therapeutic threshold for piperacillin and thrombocytopenia (ID: 9) and another with ß-lactam fever (ID: 22). Out of five patients with concentrations exceeding the upper therapeutic threshold, four had received CRRT, versus one out of seven patients who had not received CRRT (*p* = 0.021, odds ratio (OR): 15, 95% confidence interval (CI): 1 to 974; Table 2). Our analysis revealed no additional significant predictors for concentrations exceeding the upper therapeutic threshold. Neither chronic kidney disease (CKD) (*p* = 0.996, 95% CI −448 to 318) nor eGFR (*p* = 0.996, 95% CI: −3 to 653) were associated with exceeding of this threshold.

*Clearance.* In total, 13 individuals had CKD stage >2 (59%). No patients demonstrated augmented renal clearance (creatinine clearance > 130 mL/min).^33^ Among the 22 patients, 7 required CRRT. Among the 7 patients receiving CRRT, effluent rates at initiation ranged from 23 to 29 mL/kg/h. Dialysate flow rates ranged from 1000 to 1200 mL/h, and replacement fluid flow rates ranged from 600 to 1000 mL/h, with fluid removal between 0 and 100 mL/h. Data were unavailable for two patients (IDs: 2 and 4) due to missing records.

Patients had on average 0.62 mg/L lower antibiotic concentration for every 1-mL/min increase in the eGFR (*p* = 0.023, 95% CI: 1.1 to 0.092). Patients on CRRT had on average 41.2-mg/L higher antibiotic concentration than those not receiving CRRT (*p* = 0.031, 95% CI: 6.6 to 76). Albumin levels were measured for 6 patients during the collection period, with a median value of 27 g/L (interquartile range (IQR): 16–30 g/L; Table 1). The antibiotic concentration was estimated to decrease by 1.87 mg/L for every 1-g/L increase in the albumin level; however, this effect was not statistically significant (*p* = 0.634, 95% CI: −75 to 259).

## Discussion

### Main findings

The antibiotic concentration target achievement was very high among 21 ICU patients with septic shock, revealing unbound ß-lactam concentrations at or above MIC for 100% of the observation period. Additionally, augmented levels of antibiotic concentrations were common and could be related to CRRT rather than eGFR.

### Comparison with other studies

We observed an association between CRRT and augmented concentrations, contrary to the prevailing emphasis on the risk of underdosing antibiotics during CRRT in some previous studies.^34–37^ Our findings suggest that CRRT leads to elevated concentrations of ß-lactam antibiotics, potentially due to the overestimation of drug clearance, which contrasts with previous ex vivo studies demonstrating rapid antibiotic removal via CRRT circuits.^36,37^ Studies have shown that the clearance of meropenem and piperacillin–tazobactam is influenced by various CRRT modalities and prescriptions as well as effluent flow rates and residual renal function.^34,38^ Our study revealed overdosing rather than underdosing with CRRT, challenging the existing consensus on antibiotic dosing in this context. Notably, Swedish guidelines do not recommend adjusting dosage in patients with CRRT and severe infections.^39^ The significant interindividual and intra-individual variability in concentrations further complicates dosing predictions. The wide variability in drug exposure observed, independent of the prescribed dosing regimen, is consistent with previously published data. Roberts et al. have documented up to 6.7-fold variability in meropenem trough concentrations in CRRT patients receiving empirical dosing, despite relatively standardized regimens.^40^ The subsequent multinational Sampling Antibiotics in Renal Replacement Therapy (SMARRT) study confirmed this finding, demonstrating that neither the CRRT prescription nor the dosing regimen alone could reliably predict trough concentrations and that estimated total renal clearance (eTRCL) was insufficient as a sole dosing guide.^29^ The heterogeneity of CRRT practices and pathophysiology results in substantial intra- and interpatient PK variability. Our data extend these observations to the setting of extended infusion, where the relationship between dose and exposure remains similarly unpredictable, reinforcing the case for individualized TDM-guided dosing in this population. In the recently published multicenter randomized controlled trial, BLING III, no significant difference was observed in the 90-day mortality between groups receiving intermittent versus continuous infusions.^15^

### Interpretation

By visualizing the substantial PK variability observed in septic ICU patients even when antibiotics are administered via prolonged infusion, this study underscores the complex nature of hydrophilic antibiotic dosing in critically ill individuals. The presence of higher concentrations, possibly due to prolonged infusion, in many patients with acute kidney injury (AKI) and undergoing CRRT, indicates a potential overestimation of clearance. Prolonged dosing strategies may require lower doses, and it is conceivable that the combination of prolonged antibiotics and CRRT contributes to higher doses. Furthermore, the absence of augmented renal clearance in this cohort may have contributed to the high degree of target attainment observed. In this context, concentrations above the target may partly be a consequence of insufficient dose reduction rather than inherent PK variability.

This study sheds light on the issue of antibiotic toxicity. Two patients in the cohort developed adverse effects. Two patients had documented adverse effects of ß-lactam antibiotics, including one who developed thrombocytopenia and another who experienced with ß-lactam fever; both patients had received CRRT. The patient who developed thrombocytopenia exhibited intermittent episodes in which the piperacillin level exceeded the upper therapeutic target for extended infusion, indicating that the thrombocytopenia was caused by bone marrow toxicity caused by piperacillin–tazobactam. Considering these observations, the study highlights the potential value of TDM as a tool to optimize dosing and prevent toxic concentrations, particularly in patients undergoing CRRT where the actual drug clearance may be overestimated leading to augmented antibiotic concentrations.

### Strengths

One strength of this study is the frequent sampling regimen, with measurements taken every 4 h over a span of 3 days, including a comprehensive visualization of drug kinetics and the ability to evaluate time over MIC. Additionally, although this was a retrospective study, the initial samples were collected in a prospective manner, featuring broad inclusion criteria and a well-defined cohort, where 21 out of 22 patients were diagnosed with septic shock.

### Limitations

The study has certain limitations. First, this retrospective study was based on previously collected samples. In the initial HBP-KISS study, there was no sample size calculation or prespecified analysis plan as antibiotic concentration measurements were not originally intended.

Additionally, only 25% of eligible patients with signs of septic shock were enrolled. This yielded a relatively small sample size, making it challenging to draw definitive conclusions.

Furthermore, data collection and analysis were performed well after the patients were admitted and treated in the ICU, making it challenging to establish direct correlations between observations and the clinical setting. Sampling was not planned in relation to antibiotic administration, which is an inherent limitation of the post hoc study design. Finally, toxicity could not be formally assessed in this study, as trough concentrations were not available for patients on intermittent dosing, and validated steady-state toxicity thresholds remain poorly defined in the literature for patients receiving extended infusion.

## Conclusion

This small observational study indicates adequate levels of unbound ß-lactam antibiotic concentration can be achieved in most patients with septic shock using either intermittent or prolonged infusion. However, the level of concentration varies considerably between individuals and, in the same individual over time, with an associated risk of toxicity. This provides weak support for the use of TDM in clinical practice, particularly in patients undergoing CRRT.

## Supplemental Material

sj-docx-1-imr-10.1177_03000605261457800 - Supplemental material for Time over minimum inhibitory concentration and variations in beta-lactam concentrations during prolonged and intermittent infusions in patients with septic shock: A post hoc observational study Supplemental material, sj-docx-1-imr-10.1177_03000605261457800 for Time over minimum inhibitory concentration and variations in beta-lactam concentrations during prolonged and intermittent infusions in patients with septic shock: A post hoc observational study by Ylva Mattisson and Jonas Tverring in Journal of International Medical Research

## References

[1] Rudd KE Johnson SC Agesa KM , et al. Global, regional, and national sepsis incidence and mortality, 1990–2017: analysis for the global burden of disease study. Lancet 2020; 395: 200–211. 31954465 10.1016/S0140-6736(19)32989-7 PMC6970225

[2] Cecconi M Evans L Levy M , et al. Sepsis and septic shock. Lancet 2018; 392: 75–87. 29937192 10.1016/S0140-6736(18)30696-2

[3] Evans L Rhodes A Alhazzani W , et al. Surviving sepsis campaign: international guidelines for management of sepsis and septic shock 2021. Intensive Care Med 2021; 47: 1181–1247. 34599691 10.1007/s00134-021-06506-y PMC8486643

[4] Hatti M Solomonidi N Odenholt I , et al. Considerable variation of trough β-lactam concentrations in older adults hospitalized with infection-a prospective observational study. Eur J Clin Microbiol Infect Dis 2018; 37: 485–493. 29380225 10.1007/s10096-018-3194-x PMC5816762

[5] Abdul-Aziz MH Sulaiman H Mat-Nor MB , et al. Beta-lactam infusion in severe sepsis (BLISS): a prospective, two-centre, open-labelled randomised controlled trial of continuous versus intermittent beta-lactam infusion in critically ill patients with severe sepsis. Intensive Care Med 2016; 42: 1535–1545. 26754759 10.1007/s00134-015-4188-0

[6] Veiga RP Paiva JA . Pharmacokinetics–pharmacodynamics issues relevant for the clinical use of beta-lactam antibiotics in critically ill patients. Crit Care 2018; 22: 233. 30244674 10.1186/s13054-018-2155-1 PMC6151903

[7] Roberts JA Paul SK Akova M , et al. DALI: defining antibiotic levels in intensive care unit patients: are current ß-lactam antibiotic doses sufficient for critically ill patients? Clin Infect Dis 2014; 58: 1072–1083. 24429437 10.1093/cid/ciu027

[8] Ericson JE Hornik CP Greenberg RG , et al. Paradoxical antibiotic effect of ampicillin: use of a population pharmacokinetic model to evaluate a clinical correlate of the eagle effect in infants with bacteremia. Pediatr Infect Dis J 2020; 39: 725–729. 32235247 10.1097/INF.0000000000002663 PMC8628496

[9] English BK . Limitations of beta-lactam therapy for infections caused by susceptible gram-positive bacteria. J Infect 2014; 69: S5–S9. 10.1016/j.jinf.2014.07.010 25124369

[10] Roger C Louart B . Beta-lactams toxicity in the intensive care unit: an underestimated collateral damage? Microorganisms 2021; 9: 1505. 34361942 10.3390/microorganisms9071505 PMC8306322

[11] Dhaese SAM De Kezel M Callant M , et al. Emergence of antimicrobial resistance to piperacillin/tazobactam or meropenem in the ICU: intermittent versus continuous infusion. A retrospective cohort study. J Crit Care 2018; 47: 164–168. 30005302 10.1016/j.jcrc.2018.07.003

[12] Mellhammar L Wullt S Lindberg Å , et al. Sepsis incidence: a population-based study. Open Forum Infect Dis 2016; 3: ofw207. 10.1093/ofid/ofw207 PMC5144652 27942538

[13] Sime FB Roberts MS Peake SL , et al. Does Beta-lactam pharmacokinetic variability in critically ill patients justify therapeutic drug monitoring? A systemic review. Ann Intensive Care 2012; 2: 35. 22839761 10.1186/2110-5820-2-35 PMC3460787

[14] Beumier M Casu GS Hites M , et al. β-lactam antibiotic concentrations during continuous renal replacement therapy. *Crit Care* 2014; 18: R105. 10.1186/cc13886 PMC4075122 24886826

[15] Dulhunty JM Brett SJ De Waele JJ , et al. Continuous vs intermittent β-lactam antibiotic infusions in critically ill patients with sepsis: the BLING III randomized clinical trial. JAMA 2024; 332: 629–637. 10.1001/jama.2024.9779 PMC11170452 38864155

[16] Curti C Souab HK Lamy E , et al. Stability studies of antipyocyanic beta-lactam antibiotics used in continuous infusion. Pharmazie 2019; 74: 357–362. 31138374 10.1691/ph.2019.8215

[17] Tverring J Nielsen N Dankiewicz J , et al. Repeated measures of heparin-binding protein (HBP) and procalcitonin during septic shock: biomarker kinetics and association with cardiovascular organ dysfunction. Intensive Care Med Exp 2020; 8: 51. 32910266 10.1186/s40635-020-00338-8 PMC7483682

[18] Singer M Deutschman CS Seymour C , et al. The third international consensus definitions for sepsis and septic shock (sepsis-3). JAMA 2016; 315: 801–810. 26903338 10.1001/jama.2016.0287 PMC4968574

[19] von Elm E Altman D Egger M , et al. The Strengthening the Reporting of Observational Studies in Epidemiology (STROBE) statement: guidelines for reporting observational studies. Ann Intern Med 2007; 147: 573–577. 17938396 10.7326/0003-4819-147-8-200710160-00010

[20] Charlson ME Pompei P Ales KL , et al. A new method of classifying prognostic comorbidity in longitudinal studies: development and validation. J Chronic Dis 1987; 40: 373–383. 3558716 10.1016/0021-9681(87)90171-8

[21] Nyman U Grubb A Larsson A , et al. The revised Lund-Malmö GFR estimating equation outperforms MDRD and CKD-EPI across GFR, age and BMI intervals in a large Swedish population. Clin Chem Lab Med 2014; 52: 815–824. 10.1515/cclm-2013-0741 24334413

[22] Dalla Zuanna P Curci D Lucafò M , et al. Preanalytical stability of 13 antibiotics in biological samples: a crucial factor for therapeutic drug monitoring. Antibiotics (Basel) 2024; 13: 675. 39061358 10.3390/antibiotics13070675 PMC11274111

[23] Zhang YV Wei B Zhu Y , et al. Liquid chromatography-tandem mass spectrometry: an emerging technology in the toxicology laboratory. Clin Lab Med 2016; 36: 635–661. 27842783 10.1016/j.cll.2016.07.001

[24] El-Haffaf I Guilhaumou R Velly L , et al. Impact of piperacillin unbound fraction variability on dosing recommendations in critically ill patients. Br J Clin Pharmacol 2023; 89: 1502–1508. 36445340 10.1111/bcp.15619

[25] Al-Shaer MH Alghamdi WA Graham E , et al. Meropenem, cefepime, and piperacillin protein binding in patient samples. Ther Drug Monit 2020; 42: 129–132. 31318843 10.1097/FTD.0000000000000675

[26] Beijer G Clarin L Östervall J , et al. Reproducible quantification of unbound fractions of four Beta-lactam antibiotics: ultrafiltration versus microdialysis of spiked healthy donor plasma. Ther Drug Monit 2023; 45: 45–54. 35971673 10.1097/FTD.0000000000001016 PMC10321508

[27] Scharf C Paal M Schroeder I , et al. Therapeutic drug monitoring of meropenem and piperacillin in critical illness-experience and recommendations from one year in routine clinical practice. Antibiotics (Basel) 2020; 9: 131. 32245195 10.3390/antibiotics9030131 PMC7148485

[28] Tournayre S Mathieu O Villiet M , et al. Factors associated with meropenem pharmacokinetic/pharmacodynamic target attainment in septic critically ill patients treated with extended intermittent infusion or continuous infusion. Int J Antimicrob Agents 2023; 62: 106868. 37244425 10.1016/j.ijantimicag.2023.106868

[29] Roberts JA Joynt GM Lee A , et al. The effect of renal replacement therapy and antibiotic dose on antibiotic concentrations in critically ill patients: data from the multinational sampling antibiotics in renal replacement therapy study. Clin Infect Dis 2021; 72: 1369–1378. 32150603 10.1093/cid/ciaa224

[30] Imani S Buscher H Marriott D , et al. Too much of a good thing: a retrospective study of β-lactam concentration–toxicity relationships. J Antimicrob Chemother 2017; 72: 2891–2897. 29091190 10.1093/jac/dkx209

[31] Zerbib Y Gaulin C Bodeau S , et al. Neurological burden and outcomes of excessive β-lactam serum concentrations of critically ill septic patients: a prospective cohort study. J Antimicrob Chemother 2023; 78: 2691–2695. 37694500 10.1093/jac/dkad284

[32] Guilhaumou R Benaboud S Bennis Y , et al. Optimization of the treatment with beta-lactam antibiotics in critically ill patients-guidelines from the French Society of Pharmacology and Therapeutics (Société Française de Pharmacologie et Thérapeutique-SFPT) and the French Society of Anaesthesia and Intensive Care Medicine (Société Française d’Anesthésie et Réanimation-SFAR). Crit Care 2019; 23: 104. 30925922 10.1186/s13054-019-2378-9 PMC6441232

[33] Udy AA Roberts JA Boots RJ , et al. Augmented renal clearance: implications for antibacterial dosing in the critically ill. Clin Pharmacokinet 2010; 49: 1–16. 20000886 10.2165/11318140-000000000-00000

[34] Fiore M Peluso L Taccone FS , et al. The impact of continuous renal replacement therapy on antibiotic pharmacokinetics in critically ill patients. Expert Opin Drug Metab Toxicol 2021; 17: 543–554. 33733979 10.1080/17425255.2021.1902985

[35] Lewis SJ Mueller BA . Antibiotic dosing in critically ill patients receiving CRRT : underdosing is overprevalent. Semin Dial 2014; 27: 441–445. 25204875 10.1111/sdi.12203

[36] Jamal JA Udy AA Wallis SC , et al. Can we use an *ex vivo* continuous hemofiltration model to describe the adsorption and elimination of meropenem and piperacillin? Int J Artif Organs 2015; 38: 419–424. 26349527 10.5301/ijao.5000422

[37] Honeycutt CC McDaniel CG McKnite A , et al. Meropenem extraction by *ex vivo* extracorporeal life support circuits. J Extracorpor Technol 2023; 55: 159–166. 10.1051/ject/2023035 PMC10723574 38099629

[38] Jamal JA Udy AA Lipman J , et al. The impact of variation in renal replacement therapy settings on piperacillin, meropenem, and vancomycin drug clearance in the critically ill: an analysis of published literature and dosing regimens. Crit Care Med 2014; 42: 1640–1650. 24674926 10.1097/CCM.0000000000000317

[39] The Swedish Reference Group of Antibiotics. Dosing recommendations for antimicrobial drugs in renal replacement therapy (dialysis), https://forening.sls.se/media/gvudkku5/dosrekommendationer-fo-r-antimikrobiella-la-kemedel-8e-upplagan-2026.pdf (2024, accessed 6 September 2024).

[40] Roberts DM Roberts JA Roberts MS , et al. Variability of antibiotic concentrations in critically ill patients receiving continuous renal replacement therapy: a multicentre pharmacokinetic study. Crit Care Med 2012; 40: 1523–1528. 22511133 10.1097/CCM.0b013e318241e553

